# Robotic parastomal hernia repair

**DOI:** 10.1590/S1677-5538.IBJU.2020.0347

**Published:** 2021-02-03

**Authors:** Marcos Tobias-Machado, Daniel Coser Gomes, Eliney Ferreira Faria, Hamilton de Campos Zampolli

**Affiliations:** 1 Faculdade de Medicina do ABC-FMABC Santo André SP Brasil Faculdade de Medicina do ABC-FMABC, Santo André, SP, Brasil; 2 Instituto do Câncer Dr. Arnaldo Vieira de Carvalho São Paulo SP Brasil Instituto do Câncer Dr. Arnaldo Vieira de Carvalho, São Paulo, SP, Brasil; 3 Rede D'or São Luiz São Paulo SP Brasil Serviço de Urologia, Rede D'or São Luiz, São Paulo, SP, Brasil; 4 Hospital Municipal Dr. José de Carvalho Florence São José dos Campos SP Brasil Serviço de Urologia, Hospital Municipal Dr. José de Carvalho Florence, São José dos Campos, SP, Brasil; 5 Hospital do Câncer de Barretos Barretos SP Brasil Serviço de Urologia, Hospital do Câncer de Barretos, Barretos, SP, Brasil; 6 Hospital Alemão Oswaldo Cruz São Paulo SP Brasil Serviço de Urologia, Hospital Alemão Oswaldo Cruz, São Paulo, SP, Brasil

## Abstract

**Introduction and Objective:**

Annually, more than one hundred thousand new stomas are created in the United States and near 30-50% of those will develop parastomal hernia ( 1 ). Occasionally parastomal hernias may result in life threatening complications such as bowel obstruction or strangulation requiring urgent surgical intervention ( 2 ). The minimally invasive surgery for these hernias are preferred, specially when the primary case was either laparoscopic or robot-assisted. Our objective is to demonstrate a step-by-step robotic approach with and without mesh placement and their outcomes in two different scenarios: elective and emergency.

**Materials and Methods:**

We present two cases, a 56-year-old male with three years prior robot-assisted radical cystectomy with ileal conduit and a 82-year-old male with five year post operation of laparoscopic radical cystectomy with bilateral ureterostomies. Both of them had parastomal hernia, the first case was an urgency due to bowel obstruction while the second case was an elective procedure. Using three portals, we choose the primary repair for the first case and placement of a biological mesh within the keyhole technique ( 3 ) for the second one.

**Results:**

In the first case we had an operative time of 110min, total blood loss of 40cc and for the second case an operative time of 140min with total blood loss of 20cc. Both patients were discharged within 24h and had a follow-up of 2 years with no recurrence.

**Conclusions:**

The capability for complex sutures and dissection of intracorporeal structures makes the robotic platform a powerful ally ( 4 ) and we believe in its superiority over conventional laparoscopy. Although further studies are required, our initial series suggests that the robotic parastomal hernia repair is feasible and reproducible, with or without mesh placement and could be demonstrated its use for either elective or emergency situations.

**Figure:** Int Braz J Urol. 2021; 47 (Video #08): 468-9 http://www.intbrazjurol.com.br/video-section/20200347_Gomes_et_al
